# Chylomicronemia through a burr hole: A case report

**DOI:** 10.3389/fcvm.2022.1020397

**Published:** 2022-10-12

**Authors:** Wann Jia Loh, Ramesh Bakthavachalam, Tavintharan Subramaniam, Sharon Pek, Fionn Chua, Lester Lee, Gerald F. Watts

**Affiliations:** ^1^Department of Endocrinology, Changi General Hospital, Singapore, Singapore; ^2^Medical School, University of Western Australia, Perth, WA, Australia; ^3^Department of Cardiology and Internal Medicine, Royal Perth Hospital, Perth, WA, Australia; ^4^Duke-NUS Medical School, Singapore, Singapore; ^5^Department of Neurosurgery, National Neuroscience Institute, Singapore, Singapore; ^6^Diabetes Centre, Admiralty Medical Centre, Singapore, Singapore; ^7^Clinical Research Unit, Khoo Teck Puat Hospital, Singapore, Singapore; ^8^Department of Dietetics, Changi General Hospital, Singapore, Singapore

**Keywords:** lactescent subarachnoid haemorrhage, chylomicronaemia syndrome, multifactorial chylomicronaemia syndrome, polygenic chylomicronemia, purulent cerebrospinal fluid, triglyceride (TG), cerebrospinal fluid (CSF), hypertriglyceridemia

## Abstract

Chylomicronemia has either a monogenic or multifactorial origin. Multifactorial chylomicronemia is the more common form and is due to the interaction of genetic predisposition and secondary factors such as obesity, diabetes, unhealthy diet, and medications. We report a case of a 38-year-old man who was diagnosed with multifactorial chylomicronemia following presentation with a subarachnoid hemorrhage requiring emergency surgery through a burr hole; lactescent cerebrospinal fluid mixed with blood was observed through the burr hole. The serum triglyceride concentration was 52⋅4 mmol/L with a detectable triglyceride concentration in the cerebrospinal fluid. Rapid weight gain leading to obesity and related unfavorable lifestyle factors were identified as key secondary causes of chylomicronemia. Gene testing revealed a homozygous variant in *APOA5* and a heterozygous common variant in *GPIHBP1*. Accompanied with secondary causes, the interactions of gene and environmental conditions contribute to chylomicronemia. With aggressive medical treatment including excess weight loss, healthy diet, cessation of alcohol, and combination of anti-lipemic medications, normal plasma triglyceride levels were achieved.

## Background

Severe hypertriglyceridemia, a serum triglyceride level exceeding 1,000 mg/dl (≈10–11 mmol/L), typically reflects chylomicronemia (1), which may be detected visually as an opaque, lactescent appearance of the plasma. The most common cause of chylomicronemia is multifactorial chylomicronemia, a typically polygenic condition in which genetic risk factors with predisposition to impaired clearance of triglyceride rich lipoproteins, interact with obesity, unhealthy diet, and diabetes to cause a rise in plasma levels of very low density lipoprotein (VLDL) and chylomicrons (1, 2). Other causes are familial chylomicronemia syndrome and familial partial lipodystrophy (1, 2). However, majority of patients with severe chylomicronemia do not exhibit features of chylomicronemia syndrome such as abdominal pain, acute pancreatitis, and eruptive xanthomas, unless the serum triglyceride concentration remains persistently elevated above 20 mmol/L (3–5).

We present a case of acute onset of life-threatening subarachnoid hemorrhage and lactescent cerebrospinal fluid that led to the diagnosis of severe hypertriglyceridemia due to chylomicronemia. We aim to highlight the importance of heart-healthy diet and lifestyle in the management of multifactorial chylomicronemia.

## Case report

**FIGURE 1:**
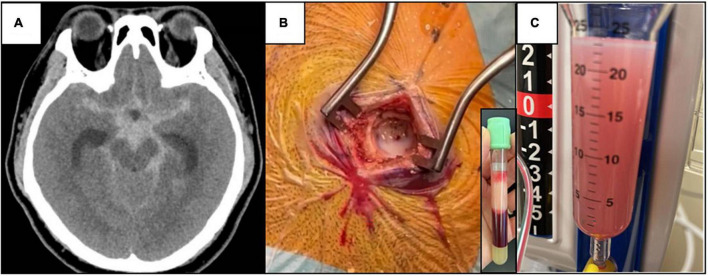
**(A)** Non-contrast CT brain showing extensive subarachnoid hemorrhage centered at the suprasellar and interpeduncular cisterns with cerebral edema. **(B)** Lactescent subarachnoid hemorrhage was seen during operation (CSF TG level of 6⋅88 mmol/L), and the corresponding lipemic blood plasma due to hypertriglyceridemia (serum TG level of 52⋅4 mmol/L). **(C)** Blood-stained turbid CSF in the tubing connected to the external ventricular drain, day 2 after the operation.

A 38-year-old man presented to the hospital with acute-onset severe headache associated with unsteady gait and dysarthria. There was no history of trauma. His Glasgow coma scale (GCS) was 14, blood pressure 203/137 mmHg, heart rate 124 beats/min, and body mass index 30 kg/m^2^. Computed tomography (CT) scanning of the brain showed an extensive acute subarachnoid hemorrhage at the suprasellar and interpeduncular cisterns, with marked cerebral edema with hydrocephalus (Figure 1A). The CT angiogram did not show aneurysms. An emergency surgery was performed. A burr hole was drilled into his skull and upon dural opening, a lactescent fluid was seen (Figure 1B), which was initially considered purulent, and cerebrospinal fluid (CSF) white cell count, Gram stain, cultures, and serum procalcitonin were subsequently normal, excluding pyogenic meningitis and brain empyema. An external ventricular drain was inserted, and a blood-stained lactescent fluid was drained into the chamber (Figure 1C).

**FIGURE 2:**
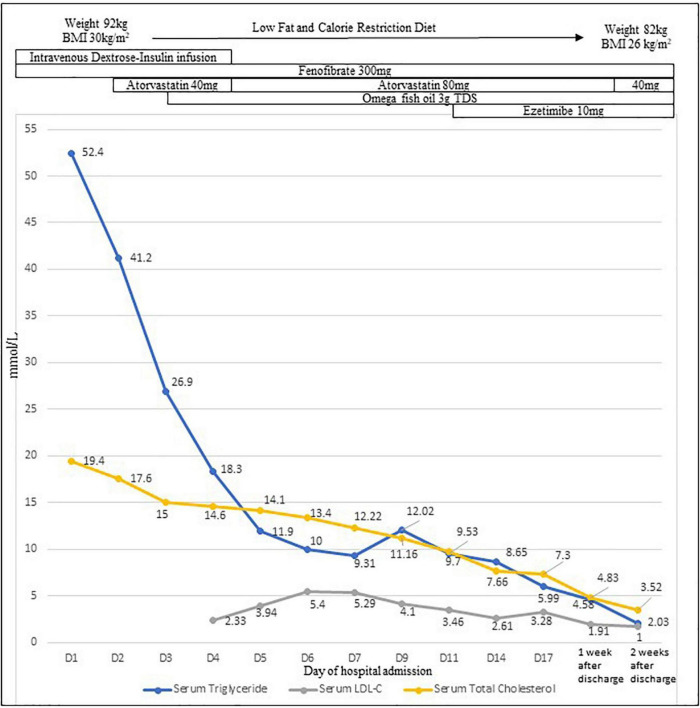
Profiles of serum triglyceride level, CSF triglyceride level, serum total cholesterol, and LDL-C levels with treatment.

The findings prompted the testing of serum lipids, which revealed a markedly elevated serum triglyceride (TG) level of 52.4 mmol/L with a paired CSF TG level of 6.8 mmol/L; total cholesterol was 17.6 mmol/L, HDL cholesterol (HDL-C) was 0.26 mmol/L, and LDL-C was unmeasurable. Despite the high triglyceride levels, he did not have signs of eruptive xanthomata and lipemia retinalis, or pancreatitis or diabetes mellitus (HbA1c 5.5%). Following endocrine review, intravenous dextrose-insulin infusion, fenofibrate, and atorvastatin were administered, with improvement in TG to <10 mmol/L on day 11 post-admission (Figure 2). CSF TG level was undetectable with a corresponding serum TG of 26.9 mmol/L on day 3, implying that the initial hypertriglyceridemic CSF was due to the spillover from the serum related to the intracranial bleeding disrupting the blood-brain barrier (BBB).

Thyroid, cortisol, renal, and coagulation studies were unremarkable. Liver enzymes were elevated (ALT 148 U/L and AST 71 U/L) but normalized after a month. Blood pressure was controlled with four anti-hypertensives, the GCS normalized, and he was extubated the day after the surgery. The patient recalled that the health screening 3 years ago showed TG > 5 mmol/L (exact value not available), which prevented the calculation of LDL-C, but he did not seek a medical review. He had gained 20 kg in weight in the past 2 years related to a sedentary lifestyle imposed by restrictions during the COVID-19 pandemic in Singapore. During this period, his diet was excessive in fat (>120 g/day) and carbohydrates (>250 g/day), and included regular intake of fatty pork, fried foods, and instant noodles. He also started drinking a bottle of vodka at weekends and two cans of beer each weekday, and he consumed one large bottle of orange juice concentrate every fortnight. He did not smoke and was not taking regular medications. Despite severe hypertriglyceridemia and excessive alcohol consumption, he never experienced pancreatitis. There was no family history of intracranial hemorrhage, aneurysm, hypertriglyceridemia, or related complications.

The testing for genetic causes of chylomicronemia in *LPL*, *APOC2*, *APOA5*, *LMF1*, and *GPIHBP1* revealed a homozygous variant, c.553G > T, p.Gly185Cy in *APOA5*, which is associated with hypertriglyceridemia (6), and a heterozygous common variant, c.41G > T, p.Cys14Phe, in *GPIHBP1.* A diagnosis of polygenic or multifactorial chylomicronemia precipitated by excessive alcohol, obesity, and poor diet was made. The transthoracic echocardiogram was normal. Secondary causes of severe hypertension including pheochromocytoma, renal failure, and renal artery stenosis were excluded. In this case, uncontrolled undiagnosed hypertension and high alcohol consumption were major risk factors for spontaneous subarachnoid hemorrhage.

Prior to discharge, the external ventricular drainage was internalized to a ventriculoperitoneal shunt because he was still shunt-dependent. During his inpatient stay, his diet plan was of a very low fat (<20 g/day) and reduced calorie (<1,200 kcal/day) diet to reduce the chylomicronemia and promote weight loss. He was discharged well with a serum TG level of 6 mmol/L (day 17). Advice on a low-fat and balanced diet, abstention from alcohol, regular exercise, and weight loss was given. Three months after the initial presentation, he successfully lost 20 kg in weight by adhering to a low-fat (<40 g/day) and calorie-restricted (<1,200 kcal/day) diet. Multivitamin was prescribed to ensure sufficient micronutrient intake. The patient’s serum TG level was 1⋅1 mmol/L and LDL-C.8 mmol/L on fenofibrate, atorvastatin, ezetimibe, and omega-3 fish oil, with blood pressure being well-controlled with dual agents. The patient reported that overall he felt more alert and energetic with the healthier diet, alcohol cessation, and excess weight loss.

## Discussion

This is a rare case report of an unusual presentation of chylomicronemia, presenting with an acute onset of severe headache due to subarachnoid haemorrhage with lactescent appearance, confirmed with measurement of elevated triglyceride concentration in the CSF. We found only two other published case reports with similar presentation; Chen et al. reported on a 49-year-old man with acute symptoms of altered consciousness and left hemiplegia due to a large intracerebral hematoma (7). The emergency evacuation of the hematoma revealed “milky”-colored cerebrospinal fluid in the external ventricle drain. The lactescent CSF was presumably due to severe hypertriglyceridemia, as the serum triglyceride was elevated at 153.67 mmol/L (13,611 mg/dl) (7). Despite best measures, the patient died (7). Another case reported was a 38-year-old male on warfarin who presented with GCS of 9 due to an acute intracerebral hemorrhage with intraventricular extension and obstructive hydrocephalus (8). The “purulent” CSF was too viscous to be drained easily *via* external ventricular drain (8). Similar to our case, the CSF was actually lactescent and not purulent, supported by absent signs of infection. The serum triglyceride level was raised at 2.6 mmol/L (2,274 mg/dl) (8). In both reported cases, the triglyceride concentrations of the CSF were not reported.

The initial hypertriglyceridemic CSF was likely a consequence of the intracranial bleeding disrupting the BBB causing an appearance of lactescent subarachnoid hemorrhage. Chylomicrons are large triglyceride-rich lipoproteins that do not to cross an intact BBB (9). Chylomicrons synthesized from dietary fat in the intestine are lipoproteins composed of cholesterol, triglyceride, apolipoprotein B48, cholesterol ester, and phospholipids, with triglyceride making up most of the chylomicron structure (10). As chylomicrons have the lowest density in all lipoproteins, they form a lipemic floating layer at the top of the plasma and, in our case, CSF blood products due to subarachnoid hemorrhage. Chylomicrons, cholesterol, and plasma lipoproteins are unable to cross the BBB, which restricts movements into and out of the central nervous system (9). Chylomicrons are unable to cross the BBB and artery wall because of its large size and hence not atherogenic, at least not directly (10). Triglycerides may be able to cross the BBB as shown in a mouse study employing radioactive tracer technique (11), but the underlying mechanism of TG transport and the maximal capacity of transport across the BBB are unknown. The concentration of TG in human CSF is markedly low (9, 11). Hence, it is very unlikely that hypertriglyceridemic serum would induce hypertriglyceridemic CSF in the presence of intact BBB in humans. In a small study on 39 people, triglycerides were present in human CSF at a very low quantity with a mean TG level 0.007 mmol/L (0.65 mg/dl), representing 0.6% of blood triglyceride levels (11). In the brain, unlike the plasma, it is apolipoprotein E (ApoE) together with apoA1 that forms HDL-like particles to redistribute cholesterol and phospholipids to neuronal cells necessary for remodeling and repair (9).

Chylomicronemia is defined as abnormal accumulation of chylomicrons in the plasma for 12–14 h after a meal, typically with TG levels > 10 mmol/L or > 1,000 mg/dL (12, 13). Chylomicronemia *syndrome* is defined as chylomicronemia in the presence of eruptive xanthomas, lipemia retinalis, recurrent abdominal pain, pancreatitis, and hepatosplenomegaly (12, 13). In our case, he did not have these symptoms, although it is debatable whether hyperviscosity itself should be recognized as part of “chylomicronemia syndrome.” Our patient’s uncontrolled undiagnosed hypertension and high alcohol consumption were the main risk factors for spontaneous subarachnoid hemorrhage. However, it is possible that hypertriglyceridemia contributed to hyperviscosity syndrome (14), which is associated with increased risk of bleeding (15) and thromboembolism including stroke (16). Hyperviscosity caused by hypertriglyceridemia was postulated as a pathogenetic mechanism causing stroke in case reports of hypertriglyceridemia secondary to propofol (17), and alcohol use (18). Although severe hypertriglyceridemia contributes to hyperviscosity of the plasma, hypertriglyceridemia, or chylomicronemia *per se* has not been proven to cause intracranial thrombosis or bleeding. Interestingly, Mendelian randomization studies did not show an association of hypertriglyceridemia with increased risk of thrombosis and bleeding but rather genetic predisposition to low triglyceride levels was associated with venous thromboembolism and haemorrhagic stroke (19). Fluctuations of platelet counts from thrombocytopenia and thrombocytosis was observed in a study on 84 patients with familial chylomicronemia from lipoprotein lipase deficiency (20), although the clinical significance of this is unclear.

A life-threatening complication of severe hypertriglyceridemia is acute pancreatitis, which is associated with a mortality rate of 2–6% (1, 13). As shown in this case with the initial serum TG > 50 mmol/L, patients with severe hypertriglyceridemia may not develop acute pancreatitis; the reason for this observation is unclear. A large retrospective study on 5,550 patients showed that only 5.4% of patients with hypertriglyceridemia ≥ 11.3 mmol/l (≥1,000 mg/dl) had pancreatitis (3), while another study in a large triglyceride database reported that 20% of patients with hypertriglyceridemia ≥ 33 mmol/L (≥3,000 mg/dl) developed acute pancreatitis (4). Another retrospective study reported that hypertriglyceridemia is unlikely to cause acute pancreatitis unless the triglyceride level is > 20 mmol/L; 15.8% of patients with hypertriglyceridemia > 20 mmol/L had a history of acute pancreatitis (5). We speculate that the risk of acute pancreatitis is related not only to the severity of but also the duration of hypertriglyceridemia, among other factors including genetic predisposition to diabetes and underlying etiology, e.g., excessive alcohol intake.

Unlike familial chylomicronemia syndrome, polygenic/multifactorial chylomicronemia syndrome is a potentially preventable condition. Familial chylomicronemia syndrome is a rare monogenic disorder due to mutations in the *LPL* gene leading to reduced levels of enzyme lipoprotein lipase, or mutation of genes encoding regulators of lipoprotein lipase, *GPIHBP1*, *APOA5*, *APOC2*, and *LMF1*. Multifactorial chylomicronemia syndrome is more common than familial chylomicronemia syndrome, affecting 1 in 600 of the population (21). Multifactorial chylomicronemia is a condition caused by the interaction of rare heterozygous variants of the mentioned genes, as well as others, that increases susceptibility to severe hypertriglyceridemia in the presence of adverse lifestyle factors and medical conditions such as obesity and diabetes. Other precipitating factors are high fat or carbohydrate diet and hypothyroidism, and medications including glucocorticoids, thiazide diuretics, beta-blockers, oral estrogen, retinoids, immunosuppressants, propofol, and antiretroviral agents (1, 5, 13). In our case, the high alcohol consumption, excessive dietary saturated fat and simple carbohydrates, and sedentary behavior related to COVID-19 restrictions were important precipitating factors to the severe chylomicronemia. The *APOA5* c.553G > T, p.Gly185Cys, also known as rs2075291, has been found to be associated with hypertriglyceridemia in Asians (6). The TT genotype is also associated with hypertriglyceridemia-induced pancreatitis (22). Functional studies showed that *APOA5* (p.Gly185Cys) may affect lipoprotein lipase-mediated VLDL hydrolysis as well as lipoprotein binding, resulting in defective triglyceride modulation (23, 24). The heterozygous variant c.41G > T in *GPIHBP1* has been associated with hypertriglyceridemia and is likely benign, as the minor allele frequency for the c.41G > T variant is 29% in East Asian population according to the Genome Aggregation Database. We postulate that impaired clearance of large triglyceride-rich lipoproteins, including chylomicrons and VLDL, may have been exaggerated by the combination of small-effect, triglyceride-raising variants in *APOA5* and possibly other polygenic variants with secondary factors of chylomicronemia in this case (13, 16). Polygenic variants also influence other cardiovascular risk factors such as hypertension and diabetes, which are commonly associated with this condition (1, 2).

This case illustrates that lifestyle modifications are essential for managing polygenic chylomicronemia and that healthy lifestyle and diet are essential in prevention of severe sequelae of chylomicronemia syndrome and complications.

## Data availability statement

The original contributions presented in this study are included in the article/supplementary material, further inquiries can be directed to the corresponding author.

## Ethics statement

Ethical review and approval was not required for the study on human participants in accordance with the local legislation and institutional requirements. The patient provided their written informed consent to participate in this study. Informed consent has been obtained from the patient for publication of the case report and accompanying images.

## Author contributions

WL drafted the manuscript. WL, GW, LL, TS, and SP were involved in data interpretation. TS and SP provided the gene report. WL, RB, LL, and FC were involved in data collection. All authors were involved in the management of the case, with the primary physicians and surgeons being WL, RB, and LL. All authors contributed to the writing of the report and approved the final version of the manuscript.

## Abbreviations

LDL-C: low density lipoprotein cholesterol
TG: triglyceride
CSF: cerebrospinal fluid
BMI: body mass index
CT: computed tomography.

## References

[1] Chait A. Hypertriglyceridemia. *Endocrinol Metab Clin North Am.* (2022) 51:539–55. 35963627 10.1016/j.ecl.2022.02.010

[2] Chait A Eckel RH. The chylomicronemia syndrome is most often multifactorial: a narrative review of causes and treatment. *Ann Intern Med.* (2019) 170:626–34. 10.7326/M19-0203 31035285

[3] Rashid N Sharma PP Scott RD Lin KJ Toth PP. Severe hypertriglyceridemia and factors associated with acute pancreatitis in an integrated health care system. *J Clin Lipidol.* (2016) 10:880–90. 10.1016/j.jacl.2016.02.019 27578119

[4] Zafrir B Jubran A Hijazi R Shapira C. Clinical features and outcomes of severe, very severe, and extreme hypertriglyceridemia in a regional health service. *J Clin Lipidol.* (2018) 12:928–36. 10.1016/j.jacl.2018.03.086 29685592

[5] Sandhu S Al-Sarraf A Taraboanta C Frohlich J Francis GA. Incidence of pancreatitis, secondary causes, and treatment of patients referred to a specialty lipid clinic with severe hypertriglyceridemia: a retrospective cohort study. *Lipids Health Dis.* (2011) 10:157. 10.1186/1476-511X-10-157 21906399 PMC3180406

[6] Qian X Li Y Liu X Li L Yang K Liu R The “T” allele of apolipoprotein A5 rs2075291 is significantly associated with higher total cholesterol and triglyceride and lower high-density lipoprotein cholesterol levels in Asians: a meta-analysis. *Nutr Res.* (2018) 56:11–22. 10.1016/j.nutres.2018.03.018 30055770

[7] Chen H-W Lin S-W Lai C-C. Milky cerebrospinal fluid and serum in hypertriglyceridemia. *QJM Monthly J Assoc Phys.* (2014) 108:77–8. 10.1093/qjmed/hcu140 24982204

[8] Gough B Greene-Chandos D. 1818: intracranial hemorrhage with hypertriglyceridemia mimicking empyema and ventriculitis. *Crit Care Med.* (2016) 44:530.

[9] Mahley RW. Central nervous system lipoproteins: ApoE and regulation of cholesterol metabolism. *Arterioscler Thromb Vasc Biol.* (2016) 36:1305–15. 27174096 10.1161/ATVBAHA.116.307023 PMC4942259

[10] Freeman MW Walford GA. Chapter 41 – Lipoprotein metabolism and the treatment of lipid disorders. 7th ed. In: Jameson JL De Groot LJ de Kretser DM editors. *Endocrinology: Adult and Pediatric.* Philadelphia, PA: W.B. Saunders (2016). p. 715–36.e717. 10.1002/clc.23055

[11] Banks WA Farr SA Salameh TS Niehoff ML Rhea EM Morley JE Triglycerides cross the blood-brain barrier and induce central leptin and insulin receptor resistance. *Int J Obes (Lond).* (2018) 42:391–7. 10.1038/ijo.2017.231 28990588 PMC5880581

[12] Leaf DA. Chylomicronemia and the chylomicronemia syndrome: a practical approach to management. *Am J Med.* (2008) 121:10–2. 18187065 10.1016/j.amjmed.2007.10.004

[13] Paragh G Németh A Harangi M Banach M Fülöp P. Causes, clinical findings and therapeutic options in chylomicronemia syndrome, a special form of hypertriglyceridemia. *Lipids Health Dis.* (2022) 21:21. 10.1186/s12944-022-01631-z 35144640 PMC8832680

[14] Rosenson RS Shott S Tangney CC. Hypertriglyceridemia is associated with an elevated blood viscosity Rosenson: triglycerides and blood viscosity. *Atherosclerosis.* (2002) 161:433–9. 11888528 10.1016/s0021-9150(01)00656-6

[15] Gertz MA. Acute hyperviscosity: syndromes and management. *Blood.* (2018) 132:1379–85. 30104220 10.1182/blood-2018-06-846816 PMC6161773

[16] Coull BM Beamer N de Garmo P Sexton G Nordt F Knox R Chronic blood hyperviscosity in subjects with acute stroke, transient ischemic attack, and risk factors for stroke. *Stroke.* (1991) 22:162–8. 10.1161/01.str.22.2.162 2003279

[17] Weiss M. Propofol-induced hypertriglyceridemia as a cause of stroke. *J Clin Lipidol.* (2017) 11:798.

[18] Inokuchi R Matsumoto A Azihara R Sato H Kumada Y Yokoyama H Hypertriglyceridemia as a possible cause of coma: a case report. *J Med Case Rep.* (2012) 6:412. 10.1186/1752-1947-6-412 23198781 PMC3520695

[19] Allara E Morani G Carter P Gkatzionis A Zuber V Foley CN Genetic determinants of lipids and cardiovascular disease outcomes: a wide-angled mendelian randomization investigation. *Circ Genom Precis Med.* (2019) 12:e002711. 10.1161/CIRCGEN.119.002711 31756303 PMC6922071

[20] Gaudet D Baass A Tremblay K Brisson D Laflamme N Paquette M Natural history (up to 15 years) of platelet count in 84 patients with familial hyperchylomicronemia due to lipoprotein lipase deficien cy. *J Clin Lipidol..* (2017). 11:797–8.

[21] Goldberg RB Chait AA. Comprehensive update on the chylomicronemia syndrome. *Front Endocrinol (Lausanne).* (2020) 11:593931. 10.3389/fendo.2020.593931 PMC7644836 33193106

[22] Pu N Yang Q Shi XL Chen W-W Li X-Y Zhang G-F Gene-environment interaction between APOA5 c.553G>T and pregnancy in hypertriglyceridemia-induced acute pancreatitis. *J Clin Lipidol.* (2020) 14:498–506. 10.1016/j.jacl.2020.05.003 32561169

[23] Huang YJ Lin YL Chiang CI Yen CT Lin SW Kao JT. Functional importance of apolipoprotein A5 185G in the activation of lipoprotein lipase. *Clin Chim Acta.* (2012) 413:246–50. 10.1016/j.cca.2011.09.045 22008704

[24] Sharma V Witkowski A Witkowska HE Dykstra A Simonsen JB Nelbach L Aberrant hetero-disulfide bond formation by the hypertriglyceridemia-associated p.Gly185Cys APOA5 variant (rs2075291). *Arterioscler Thromb Vasc Biol.* (2014) 34:2254–60. 10.1161/ATVBAHA.114.304027 25127531 PMC4178935

